# Supplementary material for: Gate-controlled suppression of light-driven proton transport through graphene electrodes
Source: Nat Commun. 2023 Oct 31;14:6932. doi: 10.1038/s41467-023-42617-4 (PMC10618495; doi:10.1038/s41467-023-42617-4)
Supplement: Supplementary file 1 — Supplementary Information [file 41467_2023_42617_MOESM1_ESM.pdf]

Supplementary Information

Gate-controlled suppression of light-driven proton transport through graphene electrodes

S. Huang[1,2†], E. Griffin[1,2†*], J. Cai[1,3], B. Xin[1,2], J. Tong[1,2], Y. Fu[1,2], V. Kravets[1], F. M. Peeters[4,5], M. Lozada-Hidalgo[1,2*]

[†] These authors contributed equally.

[1] Department of Physics and Astronomy, The University of Manchester, Manchester M13 9PL, UK

[2]National Graphene Institute, The University of Manchester, Manchester M13 9PL, UK

[3]College of Advanced Interdisciplinary Studies, National University of Defence Technology, Changsha, Hunan 410073, China

[4]Departamento de Fisica, Universidade Federal do Ceara, 60455-900 Fortaleza, Ceara, Brazil

[5]Departement Fysica, Universiteit Antwerpen, Groenenborgerlaan 171, B-2020 Antwerp, Belgium

[*]Corresponding emails: eoin.griffin@manchester.ac.uk ; marcelo.lozadahidalgo@manchester.ac.uk

This file contains:

- **Supplementary Figures 1-8**

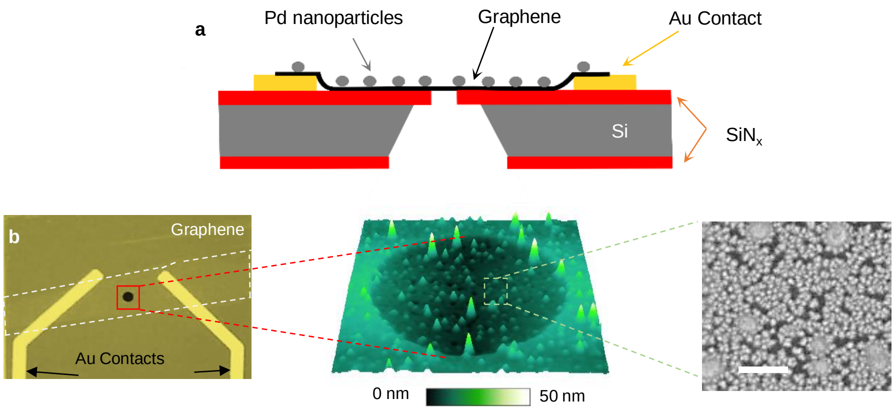

Supplementary Figure 1| Experimental devices. a, Schematic of the devices studied in this work. **b,** Top view optical image of a typical device. Black circle, 5 µm diameter aperture in the SiN_x membrane. The area covered by monolayer graphene is marked with white dashed lines. Devices had two gold electrodes to ensure good electrical contact with graphene (these were shorted). **c**, AFM image of the area marked with a red square in panel **b**. The Pd nanoparticles coalesce, resulting in discontinuous films with irregular features, evident in the image topography. These have a typical height of a couple nm with some large clusters of tens of nm. **d**, SEM of the area marked in panel **c**, which shows that the individual nanoparticles are a couple nanometres in size. Scale bar, 20 nm.

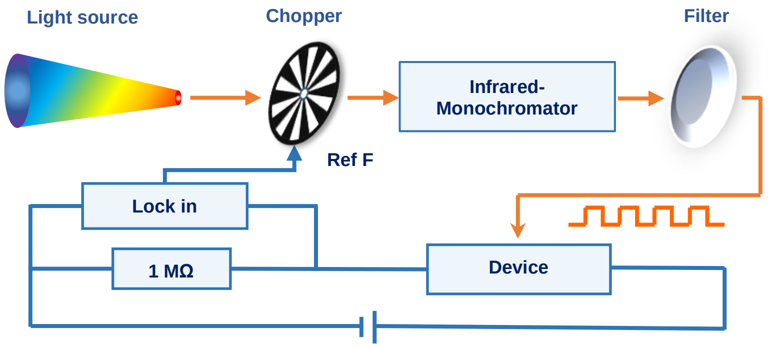

Supplementary Figure 2| Experimental setup. Experimental devices are connected into the electrical circuit shown in the figure. Monochromatic light (wavelength 1800-2500 nm) is shone through an optical chopper that functions as the reference signal for the lock-in amplifier. The transferred light is passed through an infrared monochromator and a long-pass filter to remove higher order wavelengths.

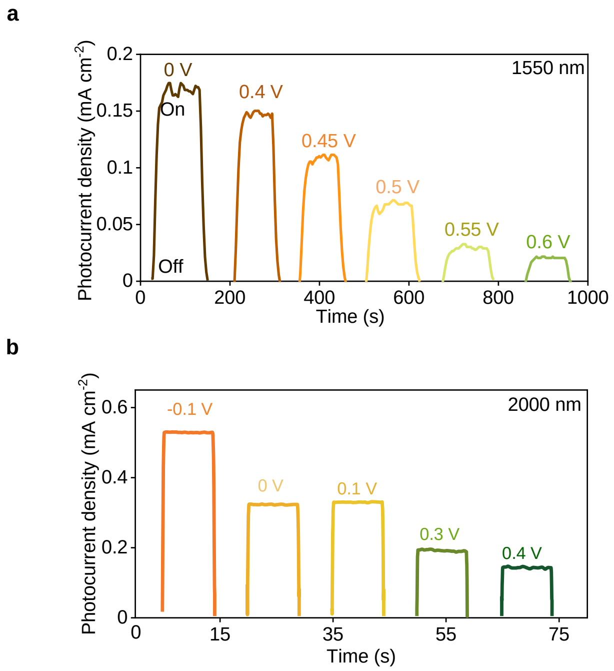

Supplementary Fig. 3| Additional examples of suppression of the photo-proton effect at constant illumination wavelength. **a**, **b**, Examples of photocurrent vs time of devices under light on-off pulses for 1550 nm and 2000 nm light, respectively.

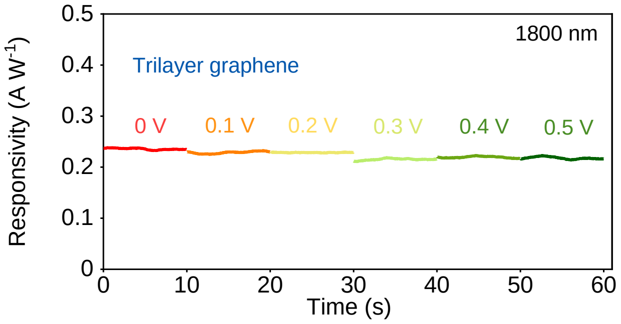

Supplementary Fig. 4| Trilayer graphene devices show voltage-independent responsivity that is an order of magnitude lower than for monolayer devices. Responsivity from a trilayer graphene device measured under illumination of 1800 nm wavelength. The data show that trilayer graphene devices have negligible responsivity that does not depend on applied bias.

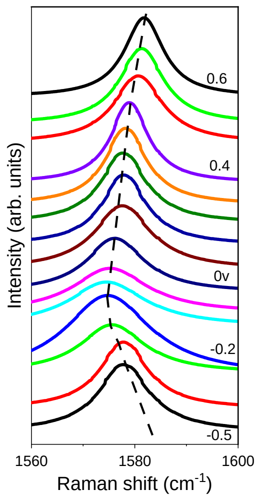

Supplementary Fig. 5| Probing Fermi energy in graphene electrodes by Raman spectroscopy. Examples of Raman spectra of the *G* band for graphene electrodes for different *V*-bias. Around 1570 cm^{-1} the band reaches a minimum, from which it blue shifts with applied bias. For clarity, the spectra are shifted along the *y*-axis. Dotted curve, guide to the eye.

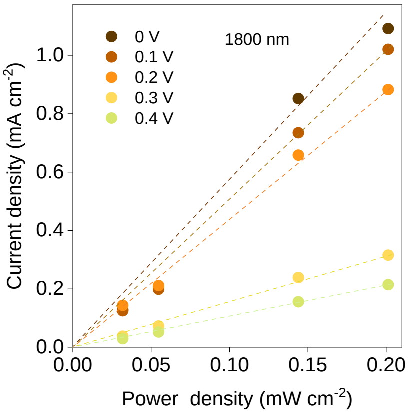

Supplementary Fig. 6| Examples of power dependence of devices' photoresponse. Examples of current density vs illumination power density for light of 1800 nm. In the measurement, the *V*-bias is fixed and the photocurrent is measured as a function of illumination power density. Dashed lines, best linear fits to data, from which we extract the photoresponsivity of the devices. The responsivity exhibits a clear drop around 0.3V – 0.4V in agreement with our measurements at constant illumination power density in Fig. 1.

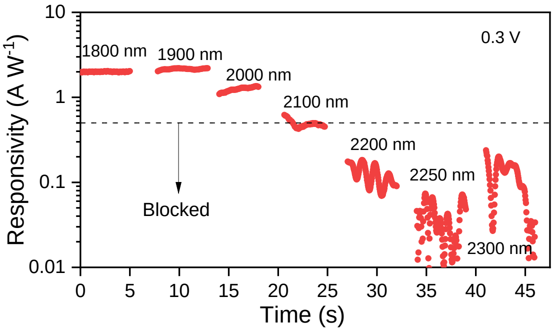

Supplementary Fig. 7| Examples of voltage-gated suppression of photoresponse at constant bias. Examples of responsivity of devices as a function of time for different wavelengths. Dashed line marks the threshold from which the devices' photoresponse is blocked.

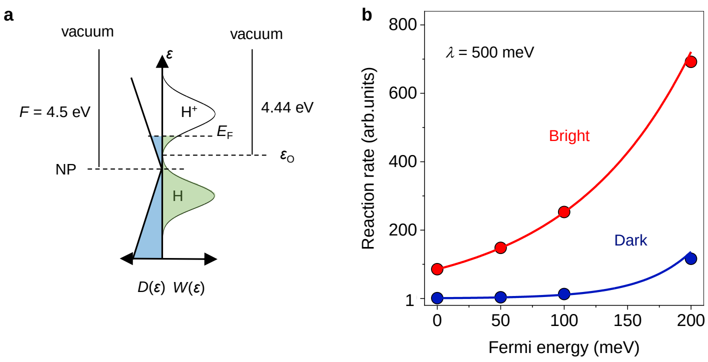

Supplementary Fig. 8| Gerischer model. a, Schematic of Gerischer model for graphene electrodes. The density of states, $D(\varepsilon)$, are filled (marked in blue) up to the Fermi level E_F. The solution states, W(ε), for the reduced (hydrogen atoms) and oxidised species (protons) are shown by two Gaussian distributions. The neutrality point (NP) and equilibrium potential for the reaction (ε_0) with respect to vacuum are known, which allows referring them with respect to each other. **b,** Reaction rate for graphene electrodes calculated using the Gerisher model for λ = 500 meV, T_e = 1000 K under illumination (300K in dark) as a function of Fermi energy in the electrode. The reaction rate is normalised against the reaction rate calculated for E_F = 0 in dark conditions.
